# Identification of Membrane Proteins in the Hyperthermophilic Archaeon *Pyrococcus Furiosus* Using Proteomics and Prediction Programs

**DOI:** 10.1002/cfg.110

**Published:** 2001-10

**Authors:** James F. Holden, Farris L. Poole II, Sandra L. Tollaksen, Carol S. Giometti, Hanjo Lim, John R. Yates III, Michael W. W. Adams

**Affiliations:** 1 Department of Biochemistry and Molecular Biology University of Georgia Athens GA 30602 USA; 2 Biosciences Division Argonne National Laboratory Argonne IL 60439 USA; 3 The Scripps Research Institute Department of Cell Biology, SR11 La Jolla CA 92037 USA; 4 Department of Biochemistry and Molecular Biology Life Sciences Building University of Georgia Athens GA 30602 USA; 5 Aventis Pharmaceuticals Bridgewater NJ 08807 USA

## Abstract

Cell-free extracts from the hyperthermophilic archaeon *Pyrococcus furiosus* were separated into membrane and cytoplasmic fractions and each was analyzed by 2D-gel electrophoresis. A total of 66 proteins were identified, 32 in the membrane fraction and 34 in the cytoplasmic fraction. Six prediction programs were used to predict the subcellular locations of these proteins. Three were based on signal-peptides (SignalP, TargetP, and SOSUISignal) and three on transmembrane-spanning α-helices (TSEG, SOSUI, and PRED-TMR2). A consensus of the six programs predicted that 23 of the 32 proteins (72%) from the membrane fraction should be in the membrane and that all of the proteins from the cytoplasmic fraction should be in the cytoplasm. Two membrane-associated proteins predicted to be cytoplasmic by the programs are also predicted to consist primarily of transmembrane-spanning *β*-sheets using porin protein models, suggesting that they are, in fact, membrane components. An ATPase subunit homolog found in the membrane fraction, although predicted to be cytoplasmic, is most likely complexed with other ATPase subunits in the membrane fraction. An additional three proteins predicted to be cytoplasmic but found in the membrane fraction, may be cytoplasmic contaminants. These include a chaperone homolog that may have attached to denatured membrane proteins during cell fractionation. Omitting these three proteins would boost the membrane-protein predictability of the models to near 80%. A consensus prediction using all six programs for all 2242 ORFs in the *P. furiosus* genome estimates that 24% of the ORF products are found in the membrane. However, this is likely to be a minimum value due to the programs’ inability to recognize certain membrane-related proteins, such as subunits associated with membrane complexes and porin-type proteins.


**Comparative and Functional Genomics**
*Comp Funct Genom* 2001; **2**: 275–288.
**DOI:** 10.1002/cfg.110

**Research Article**

### Identification of membrane proteins in the hyperthermophilic archaeon *Pyrococcus furiosus* using proteomics and prediction programs

James F. Holden[1], Farris L. Poole II[1], Sandra L. Tollaksen[2], Carol S. Giometti[2], Hanjo Lim[3]†, John R. Yates III[3] and Michael W. W. Adams[1]*

[1] *Department of Biochemistry and Molecular Biology, University of Georgia, Athens, GA 30602, USA*
[2] *Biosciences Division, Argonne National Laboratory, Argonne, IL 60439, USA*
[3] *The Scripps Research Institute, Department of Cell Biology, SR11, La Jolla, CA 92037, USA*

**Correspondence to: M. W. W. Adams, Department of Biochemistry and Molecular Biology, Life Sciences Building, University of Georgia, Athens, GA 30602, USA. E-mail: adams@bmb.uga.edu.*

*†Current address: Aventis Pharmaceuticals, Bridgewater, NJ 08807, USA.*

Received: 29 June 2001
Accepted: 20 August 2001

### Abstract

**Cell-free extracts from the hyperthermophilic archaeon *Pyrococcus furiosus* were separated into membrane and cytoplasmic fractions and each was analyzed by 2D-gel electrophoresis. A total of 66 proteins were identified, 32 in the membrane fraction and 34 in the cytoplasmic fraction. Six prediction programs were used to predict the subcellular locations of these proteins. Three were based on signal-peptides (SignalP, TargetP, and SOSUISignal) and three on transmembrane-spanning $\alpha$-helices (TSEG, SOSUI, and PRED-TMR2). A consensus of the six programs predicted that 23 of the 32 proteins (72%) from the membrane fraction should be in the membrane and that all of the proteins from the cytoplasmic fraction should be in the cytoplasm. Two membrane-associated proteins predicted to be cytoplasmic by the programs are also predicted to consist primarily of transmembrane-spanning $\beta$-sheets using porin protein models, suggesting that they are, in fact, membrane components. An ATPase subunit homolog found in the membrane fraction, although predicted to be cytoplasmic, is most likely complexed with other ATPase subunits in the membrane fraction. An additional three proteins predicted to be cytoplasmic but found in the membrane fraction, may be cytoplasmic contaminants. These include a chaperone homolog that may have attached to denatured membrane proteins during cell fractionation. Omitting these three proteins would boost the membrane-protein predictability of the models to near 80%. A consensus prediction using all six programs for all 2242 ORFs in the *P. furiosus* genome estimates that 24% of the ORF products are found in the membrane. However, this is likely to be a minimum value due to the programs' inability to recognize certain membrane-related proteins, such as subunits associated with membrane complexes and porin-type proteins. Copyright © 2001 John Wiley & Sons, Ltd.**

**Keywords:** ***Pyrococcus furiosus*; genomics; proteomics; membrane proteins; hydrophily; signal peptide; transmembrane $\alpha$-helix; beta sheet porin**

### Introduction

The advent of genome sequencing has revolutionized the study of microbial physiology. Single protein characterizations and biochemical pathway studies have been augmented by our ability to determine the functional relationships between different pathways and the roles of novel proteins. This approach, known as functional genomics, typically involves the use of DNA microarrays and proteomics (Dove, 1999; Southern *et al.*, 1999). Furthermore, insight into function may be gained from the three-dimensional structure of a protein determined by structural genomics, which involves the cloning and expression of known and unknown ORFs on a genome-wide scale and analyses of the

Copyright © 2001 John Wiley & Sons, Ltd.

product by NMR and/or X-ray crystallography (Burley, 2000).

An extremely important parameter in obtaining recombinant proteins, as well as in all aspects of both functional and structural genomics, is the subcellular location of a protein. Specifically, is the native form membrane-associated or cytoplasmic? This can be assessed in one of three ways. First, the sequences of putative proteins can be compared with those of characterized proteins of known location. However, approximately 50% of all of the predicted ORFs within 44 microbial genomes examined encode (conserved) hypothetical proteins (**http://www.tigr.org/tigr-scripts/CMR2**) and, therefore, cannot be assigned a subcellular location by simple sequence comparisons. Second, subcellular location of an ORF product may be predicted on the basis of sequence analyses for signal peptide sequences and transmembrane spanning $\alpha$-helices (Andersson *et al.*, 1992; Nielsen *et al.*, 1999). In a few cases, this has been done on a genome-wide basis. 21 genome sequences (four archaea, 14 bacteria, and three eukaryotes) were analyzed using these prediction algorithms (Kihara and Kanehisa, 2000; Mitaku *et al.*, 1999; Paulsen *et al.*, 2000; Wallin and von Heijne, 1998). They predicted that 15–30% of the ORFs in these genomes encode membrane proteins. Third, the location of proteins can be determined physically by separating cell-free extracts into cytoplasmic and membrane-associated fractions and by assessing the protein species present in each. This typically involves two-dimensional electrophoresis and the identification of proteins using mass spectrometry (Cordwell *et al.*, 2000). Separation of membrane and cytoplasmic proteins prior to proteomic analyses considerably improved the resolution and ease of identification of membrane proteins from the bacterium *Pseudomonas aeruginosa* PAO1 (Nouwens *et al.*, 2000).

Few cases can be found where more than one of the three methods used to investigate subcellular location has been used on a comparative basis to assess the validity of the results. Nouwens *et al.* (2000) used membrane protein algorithms to accurately predict *P. aeruginosa* PAO1 membrane proteins identified by proteomic methods. Hence, while there has been some success in predicting the subcellular locations of eukaryotic and bacterial proteins, investigations of the cellular location of proteins from archaeal sources are sparse and are based primarily on the characterization of purified proteins from such organisms. Understanding the architecture and physical properties of archaeal membranes is extremely important, since they differ from those of bacterial and eukaryotic membranes. In fact, Nielsen *et al.* (1999) have stated that it is unclear whether existing algorithms are adequate for predicting the subcellular locations of archaeal proteins.

To date, the genome sequences of eight hyperthermophilic (i.e., microorganisms with an optimum growth temperature above 80°C (Stetter, 1999)) archaea have been determined, three of which belong to the genus *Pyrococcus*. *Pyrococcus furiosus*, a fermentative sulfur reducer (Fiala and Stetter, 1986) whose genome sequence was recently completed (Robb *et al.*, 2001), is among the most thoroughly studied of the hyperthermophilic archaea (Adams, 1999). The relatively large biochemical literature available for *P. furiosus* together with access to the complete genome sequence suggests that this microbe could serve as a model for archaeal membrane proteins. Cytoplasmic and membrane proteins were isolated from *P. furiosus* cells and the most abundant proteins in each subcellular fraction were separated electrophoretically and identified based on peptide mass comparisons with the predicted amino acid sequences from the *P. furiosus* open reading frames (Robb *et al.*, 2001). The predicted amino acid sequences for the identified proteins were also categorized as membrane or cytoplasmic using various programs that predict signal peptides and transmembrane-spanning $\alpha$-helices. The objective was to assess their efficacy in membrane protein prediction. A consensus approach was applied genome-wide using six programs to predict the (minimum) number of membrane proteins in *P. furiosus*.

### Materials and methods

### Subcellular fractionation of proteins

*Pyrococcus furiosus* (DSM 3638) was grown in a 20-liter fermentor containing 15 liters of medium, which was prepared as described previously (Adams *et al.*, 2001; Verhagen *et al.*, 2001). The basic medium contained 0.5% (w/v) of yeast extract (Difco), casein hydrolysate (enzymatic, Difco), and maltose (Sigma); trace minerals, and the oxygen indicator resazurin in an artificial seawater solution. Where indicated, cultures also contained 0.1% (w/v) elemental sulfur (S°). Cultures were also grown on

Copyright © 2001 John Wiley & Sons, Ltd. 

the same medium except that 0.5% maltose and 0.05% yeast extract were used as the carbon sources. The headspace of the fermentor was flushed with $N_2$-$CO_2$ (80 : 20), and L-cysteine-HCl·$H_2O$ and $Na_2S·9H_2O$ were added as reducing agents to remove residual $O_2$. The pH (measured at room temperature) was adjusted to 6.8 and maintained at 5.9 $\pm$ 0.1 and 95°C during the incubation.

Cells were harvested in the late-logarithmic phase ($1\times10^8$ to $2\times10^8$ cells·$ml^{-1}$) and were cooled to room temperature by pumping them through a glass cooling coil bathed in an ice-water slurry. They were harvested by centrifugation at 10,000 × *g*, resuspended in 15 to 20 ml of Buffer A (degassed 50 mM Tris-HCl (pH 8.0) plus 2 mM sodium dithionite and 2 mM dithiothreitol), and frozen under Ar at −80°C. All sample transfers and manipulations were carried out in an anaerobic chamber and all buffers were degassed and flushed with Ar. The cell suspension was thawed, and DNase I in Buffer A was added to a final concentration of 0.0002% (w/v). The cells were disrupted anaerobically by sonication for 30 min. Debris and unbroken cells were removed by centrifugation (10,000 × *g* for 15 min), and the supernatant was decanted and centrifuged at 100,000 × *g* for 45 min. The supernatant was used as the cytoplasmic protein fraction. The membrane pellet was suspended in Buffer A, homogenized using a glass tissue grinder, and then centrifuged at 100,000 × *g* for 45 min. This procedure was repeated three times, and Buffer A in the final step contained 4 M KCl. The washed membrane pellet was suspended and homogenized in Buffer A, and this formed the membrane protein fraction. All protein fractions were then frozen in liquid $N_2$ and stored at −80°C.

Glutamate dehydrogenase (GDH) activity was determined spectrophotometrically by the reduction of 0.4 mM $NADP^+$ measured at 340 nm ($\varepsilon = 6{,}220$ $(M·cm)^{-1}$) and 80°C in 100 mM EPPS buffer (pH 8.4) using 6 mM sodium glutamate as the substrate (Robb *et al.*, 1992). The protein concentration of each fraction was estimated using the Bradford method (Bradford, 1976) with bovine serum albumin as a standard.

### Two-dimensional electrophoretic protein separation

Cytoplasmic and membrane protein samples were prepared for two-dimensional gel electrophoresis by mixing thawed fractions with an equal volume of a solution containing 9 M urea, 2% (v/v) 2-mercaptoethanol, 2% ampholytes (pH 8-10, Bio-Rad; v/v), 4% (v/v) Nonidet P40, and protease inhibitors (Complete Mini Protease Inhibitor Cocktail, Boeringer Mannheim). The samples were spun at 435,000 × *g* for 10 min (TL100 tabletop ultracentrifuge, Beckman) to remove debris. Protein concentrations of the decanted supernatant were determined using the Ramagli modification of the Bradford protein assay (Ramagli *et al.*, 1985).

First-dimension isoelectric focusing (IEF) was carried out using a 1 : 7 mixture of pH 3–10 and pH 5–7 carrier ampholytes (Bio-Rad) in 18-cm tube gels with a diameter of 1.5 mm for 14 000 V-h, as described previously (Anderson and Anderson, 1978a). Aliquots containing 20 μg of protein for silver staining or 300 μg of protein for Coomassie Blue staining were loaded onto each IEF gel. After IEF, the gels were equilibrated in a buffer containing sodium dodecyl sulfate (SDS) as described by O'Farrell (1975) and then loaded onto 10–17% polyacrylamide gradient slab gels (Anderson and Anderson, 1978b). The second-dimension separation was performed using the Laemmli buffer system (O'Farrell, 1975). The proteins were then fixed and stained in the gels using 0.2% (w/v) Coomassie blue R-250 in 2.5% phosphoric acid and 50% (v/v) ethanol (Giometti *et al.*, 1987), or fixed in 50% (v/v) ethanol with 0.1% formaldehyde and 1% acetic acid for subsequent staining with silver nitrate (Giometti *et al.*, 1991).

### Mass-spectrometric protein identification

Matrix-assisted laser desorption/ionization time-of-flight (MALDI-TOF) mass spectrometry and capillary liquid chromatography-electrospray tandem mass spectrometry (μ-LC-ESI-MS/MS) were used to identify cytoplasmic and membrane *P. furiosus* proteins extracted from 2DE gels. Protein spots to be identified were cut from 1 to 4 replicate gels stained with Coomassie Blue R250 depending on the abundance of individual proteins. The excised spots were then reduced with either dithiothreitol (Sigma) at 60°C or tris(2-carboxyethyl)phosphine (Pierce, Rockport, IL) at room temperature, alkylated with iodoacetamide (Sigma), and digested *in situ* overnight with sequencing grade modified porcine trypsin (Promega Corp., 12.5 ng/μl). The digested peptides were extracted three times with equal parts of 25 mM ammonium bicarbonate and acetonitrile and then twice with equal parts of 5%

Copyright © 2001 John Wiley & Sons, Ltd.

(v/v) formic acid and acetonitrile. The resulting extracted tryptic peptides were used directly for μ-LC-ESI-MS/MS without further purification, but were desalted and concentrated with commercial ZipTip $C_{18}$ pipette tips (Millipore, Bedford, MA) prior to MALDI-TOF peptide mass mapping analysis. Proteins were identified and confirmed by analyzing a same tryptic peptide sample with both MALDI-TOF peptide mass mapping and μ-LC-ESI tandem mass spectrometry followed by database searching.

For MALDI-TOF peptide mass mapping, tryptic digest samples, mixed with α-cyano-4-hydroxycinnamic acid (Sigma), were spotted onto a MALDI target plate and then transported into a Voyager DE-STR MALDI-TOF mass spectrometer equipped with delayed extraction and reflectron (PE Biosystem, Framingham, MA). MALDI-TOF mass spectra for each sample spot were generated by averaging 64–126 $N_2$ laser shots. Proteins were then identified using PROQUEST, a peptide mass mapping database search algorithm developed in Yates Laboratory, by comparing experimentally obtained mass-to-charge (m/z) values with theoretically calculated m/z values of tryptic peptides of proteins from a *P. furiosus* open reading frame database (**http://comb5-156.umbi.umd.edu**, GeneMate).

For μ-LC-ESI tandem mass spectrometry, tryptic digests were directly loaded onto a 10–15 cm 365 × 100 μm fused silica capillary (FSC) column packed with 10 μm POROS 10 R2 reverse-phase packing material (PE Biosystem, Framingham, MA) by using a helium-pressurized stainless-steel bomb (Gatlin *et al.*, 1998). The tryptic peptides on the column were separated in thirty minutes by performing liquid chromatography employing the linear gradient of 2–60% solvent B (A: 0.5% acetic acid, B: 80% acetonitrile/0.5% acetic acid), and then introduced to LCQ ion trap mass spectrometer (Finnigan MAT, San Jose, CA). The flow rate at the tip of 200–300 nl/min was maintained during the liquid chromatography by using a precolumn splitter. Tandem mass spectra were automatically acquired in data-dependent mode during the 30-min LC-MS runs by picking three most abundant ions above predefined threshold intensity from previous full MS scan. Obtained MS/MS spectra were then directly searched against a *P. furiosus* open reading frame database with SEQUEST database search algorithm (Eng *et al.*, 1994) without need of prior manual MS interpretation. SEQUEST identified proteins by correlating experimentally obtained MS/MS spectra to protein sequences in the *P. furiosus* database (Link *et al.*, 1997) and the identified proteins were further verified by manually checking every sequence matched with high cross-correlation scores by SEQUEST.

### Membrane protein prediction models

The predicted amino acid sequences for the ORFs identified through proteomics and in the complete genome (Robb *et al.*, 2001) were used to assess the accuracy of various membrane protein prediction programs. The programs are SignalP v1.1 (**http://www.cbs.dtu.dk/services/SignalP**; Nielsen *et al.*, 1997), TargetP v1.0 (**http://www.cbs.dtu.dk/services/TargetP**; Emanuelsson *et al.*, 2000), TSEG (**http://www.genome.ad.jp/SIT/tseg.html**; Kihara *et al.*, 1998), SOSUI and SOSUIsignal (**http://sosui.proteome.bio.tuat.ac.jp/cgi-bin/sosui.cgi**?; Hirokawa *et al.*, 1998), and PRED-TMR2 (**http://o2.db.uoa.gr/PRED-TMR2**; Pasquier and Hamodrakas, 1999). SignalP, TargetP, and SOSUISignal are based on the presence of a membrane-anchor/secretory signal peptide sequence at the N-terminus. TSEG, SOSUI, and PRED-TMR2 predict the locations of transmembrane α-helices in the peptide sequence using hydropathy models and homologies to known transmembrane α-helices, and exclude hydrophobic segments associated with globular proteins. The default settings were used for each program, except TSEG where the '5 discriminant functions' setting was used. The SOSUISignal program was run using both 'eukaryote' and 'prokaryote' settings. The ORF product was manually designated as either membrane or cytoplasmic based on the consensus results of the various programs. An ORF product was designated as membranous if at least three of the six membrane protein prediction programs yielded a positive result for that ORF.

The amino acid sequences were also analyzed using MacVector v7.0 software (Oxford Molecular Group, Symantec Corporation). With this program, the amino acid composition was determined and predictions of structure were made using plots of Kyte/Doolittle hydrophobicity (window = 7), von Heijne transmembrane (window = 21), amphiphilic helix (window = 21), amphiphilic sheet (window = 7), and Robson-Garnier secondary structure (window = 7).

Copyright © 2001 John Wiley & Sons, Ltd. 

### Genome-wide membrane protein estimations

For a genome-wide estimate of membrane-encoding ORFs, each *P. furiosus* protein sequence was run through each of the six membrane-protein prediction web sites. In order to accomplish this the analysis was broken down into three main steps: download & pre-processing, sequence submission and result parsing. In the first step, the *P. furiosus* genome sequence was download in FASTA format from GeneMate and this file was used to produce six more FASTA files that were pre-processed according to each web site's instructions. Two methods were used for sequence submission since the format of the web sites varied. The first method (SOSUISignal, TSEG, SOSUI and PRED-TMR2) relied on a web browser automation program written using Microsoft Visual Basic 6 (VB). The program was designed to submit one sequence at a time to the web site through the browser and wait for a response. Once the program received the requested web page (the results) the information on the page was automatically parsed and saved to a text file for later analysis. In the second method (SignalP and TargetP), each site's e-mail server was utilized where large blocks of the genome were submitted at a time. The returned e-mail results were parsed and saved to a text file using another program written in VB. All six result files were then imported into a Microsoft Excel spreadsheet to aid in calculation. A final prediction of protein cellular location was based on a manual consensus analysis of the results for the six membrane protein models, as described above.

### Results

### Proteins identified by proteomics

Membrane and cytoplasmic fractions were prepared from *P. furiosus* cells grown on 0.5% (w/v) each of maltose, yeast extract, and casein hydrolysate with and without S° and from cells grown on 0.5% maltose and 0.05% yeast extract without S°. For the culture grown with casein but without S°, the glutamate dehydrogenase (GDH) activities in the cytoplasmic and membrane fractions were 85% and <0.1%, respectively, of the activity in unfractionated cell-free extract. The cytoplasmic and membrane fractions contained 59 and 9%, respectively, of the total protein present in the extract. For the culture grown with casein and S°, cytoplasmic and membrane fraction GDH activities were 58 and 0.2%, respectively, of the activity in the cell-free extract, and 45 and 7% of the total protein was recovered in the cytoplasmic and membrane protein fractions, respectively. The cytoplasmic and membrane fractions from cells grown with maltose, no casein and without S° had GDH activities that were 81 and 0.2%, respectively, of the total activity in the cell-free extract, with 62 and 17% of the total protein recovered in the cytoplasmic and membrane fractions, respectively.

A total of 25 proteins from the membrane fraction of cells grown with casein and without S° were identified using two-dimensional gel electrophoresis to separate the proteins and mass spectrometry of tryptic peptides to identify their corresponding coding sequence in the open reading frame database (Figure 1). A total of 12 proteins from the membrane fraction of cells grown with S° were identified, producing a total of 32 unique proteins (5 proteins were present in both samples: Table 1). Of these 32 proteins, one had been characterized previously from *P. furiosus*, 13 were conserved hypothetical proteins, and the remaining 18 were homologs of characterized proteins. At least three of these 32 proteins (Pf336067, Pf1459639, and Pf1825269) were also found in the cytoplasmic fraction or show homology to known cytoplasmic proteins, suggesting that they could be cytoplasmic contaminants in the membrane preparations. The most abundant membrane proteins found on the gels were maltose- and dipeptide-binding protein homologs (Figure 1). A total of 34 proteins were identified in the cytoplasmic fraction (Table 2) and 8 of these were enzymes characterized previously from *P. furiosus.* Of the remainder, 11 were conserved hypothetical proteins, one was a unique hypothetical protein, and the other 14 were homologs of characterized proteins. For each fraction, all of the proteins identified using MALDI-TOF were likewise identifed using μ-LC-MS/MS. However, more proteins were identified using the latter method due to the increased sensitivity of the procedure.

### Predictability of membrane and cytoplasmic proteins

The signal-peptide recognition programs (SignalP, TargetP, and SOSUISignal) predicted that 17 to 20 of the proteins (59–69%, when excluding the three predicted cytoplasmic contaminants) identified in

Copyright © 2001 John Wiley & Sons, Ltd.

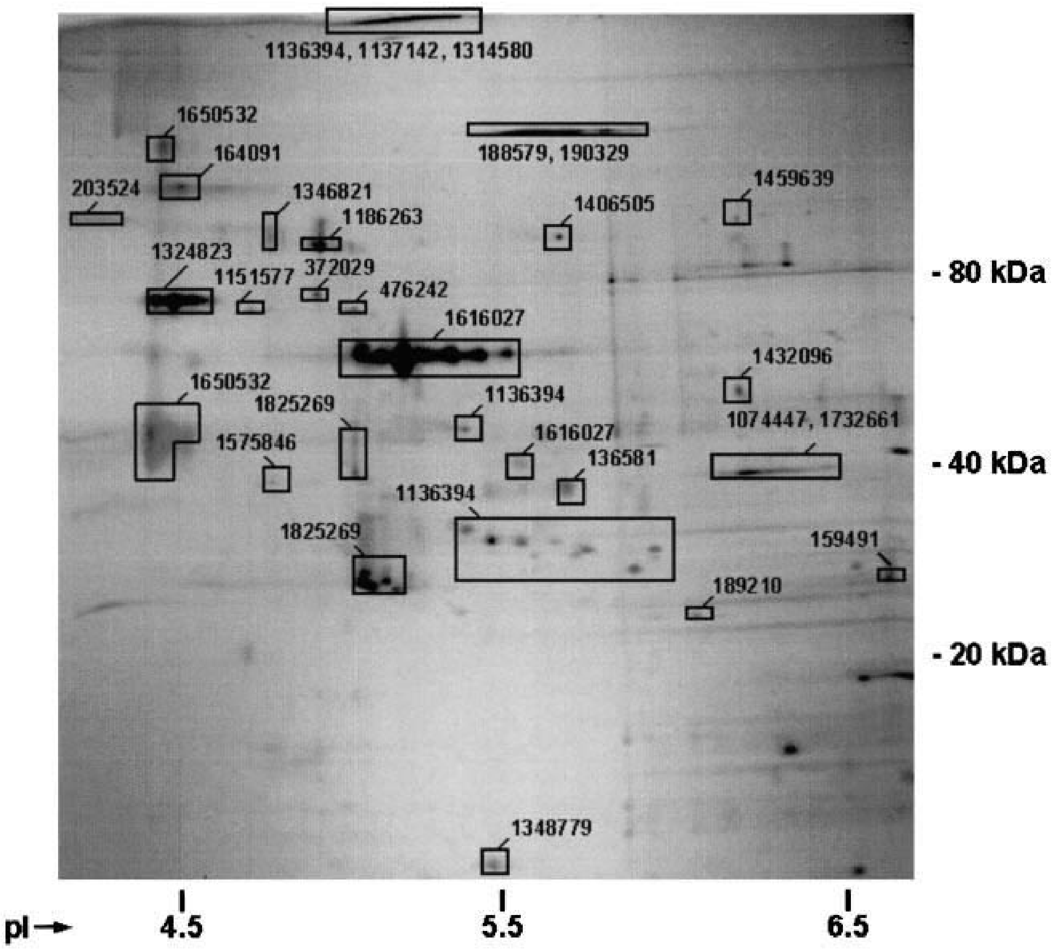

**Figure 1.** Two-dimensional gel electrophoresis pattern of membrane proteins isolated from *P. furiosus* grown without sulfur. Proteins were stained with Coomassie Blue R250. Numbers shown refer to the open reading frame (ORF) designation from the *P. furiosus* genome sequence (Robb *et al.*, 2001). Table 1 shows the annotation for the ORFs indicated the membrane fraction contained a signal peptide (Table 1). The transmembrane α-helix recognition programs (TSEG, SOSUI, and PRED-TMR2) predicted that 13 to 23 of these proteins (45–79%) contained at least one transmembrane α-helix. A consensus of the six programs predicted that 23 of these proteins (79%) were in the membrane. The signal-peptide recognition programs predicted that 31 to 34 of the proteins (91–100%) identified in the cytoplasmic fraction did not contain a signal peptide (Table 2). The transmembrane-segment recognition programs predicted that 32 to 33 of these proteins (94–97%) did not contain at least one transmembrane α-helix. A consensus of the six programs predicted that all 34 of these proteins were cytoplasmic. A chi-squared statistical test was performed on the protein location predictions. For both the membrane and cytoplasmic fractions the predictions were significant (i.e., not random; $0.01 < p < 0.025$ and $p < 0.005$, respectively).

For membrane-associated proteins that were predicted to be cytoplasmic by the above programs, additional analyses were made using MacVector v7.0 sequence analysis software. Two of the nine proteins (Pf159491 and Pf1871822) are predicted to consist primarily of hydrophobic residues with a high degree of β-sheet structure (Figure 2). The Pf1871822 protein contains high concentrations of alanine (11.7%), proline (11.1%) and glycine (15.0%), and the Pf159491 protein contains moderately high concentrations of the same residues (5.9, 3.2, and 15.0%, respectively). The remaining 7

Copyright © 2001 John Wiley & Sons, Ltd. 

Table 1. Proteins identified by proteomics in the membrane cellular fraction, and their predicted locations based on signal peptide and transmembrane sequence models

| | | | | Signal Peptide | | | Pred. # of Transmem. Seg. | | | |
|---|---|---|---|---|---|---|---|---|---|---|
| **ORF #** | ***Pyrococcus furiosus* Genome Annotation** | **Closest Characterized Homolog (except for conserved hypotheticals)** | **Id./Sim. (%/%)** | **SignalP** | **TargetP** | **SOSUI** | **TSEG** | **SOSUI** | **PRED-TMR2** | **Consensus Pred.** |
| 123510 | Putative periplasmic sugar binding protein | *Thermococcus litoralis* AAC38136 | 19/32 | Yes | Yes | Yes | 1 | 1 | 0 | Mem. |
| 136581 | Conserved hypothetical protein | *Pyrococcus abyssi* F75209 | 37/51 | Yes | Yes | No | 1 | 1 | 1 | Mem. |
| 159491 | Conserved hypothetical protein | *Pyrococcus abyssi* H75211 | 78/88 | No | No | No | 0 | 0 | 0 | Cyto. |
| 164091 | Oligosaccharyl transferase SST3 subunit related protein | *Saccharomyces cerevisiae* BAA06079 | 13/27 | Yes | No | No | 10 | 14 | 14 | Mem. |
| 188579 | ATPase subunit K | *Desulfurococcus* sp. SY AAB64412 | 45/56 | Yes | Yes | Yes | 2 | 4 | 4 | Mem. |
| 189210 | ATPase subunit E | *Desulfurococcus* sp. SY AAB64413 | 59/76 | No | No | No | 0 | 0 | 0 | Cyto. |
| 190329 | ATPase subunit C | *Desulfurococcus* sp. SY AAB64414 | 55/73 | No | Yes | Yes | 1 | 1 | 0 | Mem. |
| 203524 | Conserved hypothetical protein | *Pyrococcus horikoshii* BAA31089 | 81/90 | Yes | Yes | Yes | 2 | 3 | 2 | Mem. |
| 336067 | Related to inosine monophosphate dehydrogenase | *Methanococcus jannaschii* Q59011 | 17/32 | No | No | No | 0 | 0 | 0 | Cyto. |
| 372029 | Dipeptide-binding protein | *Bacillus subtilus* AAA62358 | 19/31 | Yes | Yes | Yes | 2 | 5 | 2 | Mem. |
| 476242 | Conserved hypothetical protein | *Pyrococcus abyssi* B75005 | 44/61 | Yes | Yes | Yes | 1 | 1 | 0 | Mem. |
| 1074447 | Conserved hypothetical protein | *Archaeoglobus fulgidus* F69482 | 44/56 | No | No | No | 0 | 0 | 0 | Cyto. |
| 1136394 | Conserved hypothetical protein | *Aquifex aeolicum* AAC07600 | 22/40 | No | No | No | 0 | 0 | 0 | Cyto. |
| 1137142 | Conserved hypothetical protein | *Pyrococcus abyssi* A75157 | 48/63 | No | No | No | 0 | 0 | 0 | Cyto. |
| 1151577 | Oligopeptide ABC transporter (oligopeptide-binding protein) | *Bacillus subtilus* P42061 | 20/33 | Yes | Yes | Yes | 2 | 2 | 1 | Mem. |
| 1186264 | Conserved hypothetical protein | *Pyrococcus abyssi* D75122 | 30/40 | No | No | No | 1 | 1 | 1 | Mem. |
| 1314580 | Putative ATPase, vanadate-sensitive | *Methanococcus voltae* A38542 | 22/37 | Yes | Yes | Yes | 2 | 1 | 0 | Mem. |
| 1324824 | Probable dipeptide-binding protein | *Escherichia coli* P23847 | 19/31 | Yes | Yes | Yes | 1 | 1 | 0 | Mem. |
| 1346821 | Conserved hypothetical protein | *Pyrococcus horikoshii* E71018 | 75/85 | Yes | Yes | Yes | 1 | 1 | 0 | Mem. |
| 1348779 | Conserved hypothetical protein | *Methanococcus jannaschii* F64490 | 36/53 | No | No | Yes | 2 | 3 | 2 | Mem. |
| 1406506 | Conserved hypothetical protein | *Pyrococcus horikoshii* BAA30439 | 52/71 | Yes | Yes | Yes | 2 | 2 | 2 | Mem. |
| 1432096 | Stomatin homolog | *Rhizobium etli* AAA84990 | 34/51 | Yes | Yes | Yes | 5 | 5 | 7 | Mem. |
| 1459639 | DNA directed RNA polymerase subunit B | *Thermococcus celer* P31814 | 84/91 | No | No | No | 0 | 0 | 0 | Cyto. |
| 1575846 | Hypothetical lipoprotein | *Borrelia burgdorferi* CAA65879 | 24/37 | Yes | Yes | No | 1 | 2 | 0 | Mem. |
| 1616027 | Trehalose/maltose binding protein (MalE) | *Thermococcus litoralis* AAC38136 | 93/93 | Yes | Yes | Yes | 1 | 2 | 0 | Mem. |
| 1650532 | Iron (III) ABC transporter, ATP-binding protein (HemV-2) | *Vibrio anguillarum* P11460 | 26/44 | Yes | Yes | Yes | 1 | 1 | 1 | Mem. |
| 1732661 | O-linked GlcNAc transferase | Rat P56558 | 7/12 | Yes | Yes | No | 4 | 4 | 4 | Mem. |
| 1783837 | Amylopullulanase | *Pyrococcus furiosus* AF016588 | 100/100 | Yes | Yes | Yes | 1 | 0 | 0 | Mem. |

Copyright © 2001 John Wiley & Sons, Ltd.

Table 1. Continued

| ORF # | *Pyrococcus furiosus* Genome Annotation | Closest Characterized Homolog (except for conserved hypotheticals) | Id./Sim. (%/%) | Signal Peptide | | | Pred. # of Transmem. Seg. | | | Consensus Pred. |
|---|---|---|---|---|---|---|---|---|---|---|
| | | | | SignalP | TargetP | SOSUI | TSEG | SOSUI | PRED-TMR2 | |
| 1788984 | Putative sugar binding protein (MalE-like) | *Salmonella typhimurium* P19576 | 28/45 | Yes | Yes | No | 1 | 1 | 0 | Mem. |
| 1817174 | Conserved hypothetical protein | *Pyrococcus horikoshii* F71220 | 84/91 | Yes | Yes | Yes | 1 | 1 | 1 | Mem. |
| 1825269 | Thermosome, single subunit | *Desulfurococcus* sp. SY AAB35235 | 87/90 | No | No | No | 0 | 0 | 0 | Cyto. |
| 1871822 | Conserved hypothetical protein | *Pyrococcus abyssi* D75078 | 65/74 | No | No | No | 0 | 0 | 0 | Cyto. |
| | | | Totals[a]: | 20 (69.0%) | 20 (69.0%) | 17 (58.6%) | 23 (79.3%) | 22 (75.9%) | 13 (44.8%) | 23 (79.3%) |

[a]Three of the 32 proteins from the membrane fraction are presumed to be cytoplasmic contaminants and were not included for the percentage calculation.

proteins from the membrane fraction identified as "cytoplasmic" had low concentrations of hydrophobic residues.

### Genome-wide estimate of membrane proteins

The SignalP, TargetP and SOSUISignal programs predicted that 389 (17%), 465 (21%) and 270 (12%), respectively, of the ORFs in the *P. furiosus* genome encode for proteins that contain a membrane target signal. The TSEG, SOSUI and PRED-TMR2 programs predict that 563 (25%), 628 (28%) and 452 (20%), respectively, of the ORFs encode for proteins that contain at least one transmembrane $\alpha$-helix. Consensus predictions using the results from all six programs suggest that 533 (24%) of the ORFs in *P. furiosus* encode proteins located in the membrane.

### Discussion

The near absence of glutamate dehydrogenase activity, a major cytoplasmic protein in *P. furiosus* (Robb *et al.*, 1992), in the membrane fraction suggests that the method of subcellular fractionation is effective. Our results indicate that between 7 and 17% of the total cellular protein is located in the membrane fraction of *P. furiosus*, depending on the growth conditions. For the proteins identified in the membrane fraction, the consensus of the signal peptide and transmembrane $\alpha$-helix programs predicted that $\geq 79\%$ should be membrane proteins. For the proteins identified from the cytoplasmic fraction, the programs predicted that all are cytoplasmic. Those proteins in the membrane fraction that were not predicted to be associated with the membrane may 1. be composed of transmembrane $\beta$-sheets rather than $\alpha$-helices, 2, form complexes with other proteins that are in the membrane, 3. represent cytoplasmic contaminants, or 4. be novel membrane proteins. At present, we have no suitable method to distinguish between these possibilities. In a genome-wide analysis using the six programs, it is estimated that 533 ORFs (24%) in the genome of *P. furiosus* encode for membrane proteins, although, based on the sequence analyses of the proteins identified by proteomics, this is may underestimate the actual number of membrane proteins by up to 20%.

Eight proteins from *P. furiosus* whole cell lysates were previously identified based on co-migration

Copyright © 2001 John Wiley & Sons, Ltd.

Table 2. Proteins identified by proteomics in the cytoplasmic cellular fraction, and their predicted locations based on signal peptide and trans-membrane sequence models

| ORF # | *Pyrococcus furiosus* Genome Annotation | Closest Characterized Homolog (except for conserved hypotheticals) | Id./Sim. (%/%) | Signal Peptide: SignalP | Signal Peptide: TargetP | Signal Peptide: SOSUI | Pred. # of Transmem. Seg.: TSEG | Pred. # of Transmem. Seg.: SOSUI | Pred. # of Transmem. Seg.: PRED-TMR2 | Consensus Pred. |
|---|---|---|---|---|---|---|---|---|---|---|
| 209264 | Conserved hypothetical protein | *Pyrococcus horikoshii* BAA31083 | 89/93 | No | No | No | 0 | 0 | 0 | Cyto. |
| 232621 | Enolase (2-phosphoglycerate dehydratase) | *Bacillus subtilus* P37869 | 59/71 | No | No | No | 0 | 0 | 0 | Cyto. |
| 275295 | 2-amino-3-ketobutyrate CoA ligase (glycine acetyl transferase) | *Bacillus subtilus* CAB13573 | 47/67 | No | No | No | 0 | 0 | 0 | Cyto. |
| 283928 | Alpha-amylase | *Pyrococcus furiosus* AAA72035 | 99/99 | No | No | No | 0 | 0 | 0 | Cyto. |
| 327695 | ADP-dependent glucokinase | *Pyrococcus furiosus* AAD48401 | 100/100 | No | No | No | 0 | 0 | 0 | Cyto. |
| 336067 | Related to inosine monophosphate dehydrogenase | *Methanococcus* jannaschii Q59011 | 17/32 | No | No | No | 0 | 0 | 0 | Cyto. |
| 392566 | Conserved hypothetical protein | *Pyrococcus abyssi* CAB50420 | 96/98 | No | No | No | 0 | 0 | 0 | Cyto. |
| 518483 | Conserved hypothetical protein | *Pyrococcus horikoshii* BAA29892 | 91/95 | No | No | Yes | 0 | 0 | 0 | Cyto. |
| 630041 | Conserved hypothetical protein | *Pyrococcus horikoshii* BAA29833 | 62/73 | No | No | No | 0 | 0 | 0 | Cyto. |
| 635169 | Conserved hypothetical protein | *Pyrococcus abyssi* CAB50211 | 98/99 | No | No | No | 0 | 0 | 0 | Cyto. |
| 703868 | Flavoprotein | *Mb. thermoautotrophicum* S66533 | 34/53 | No | No | No | 0 | 0 | 0 | Cyto. |
| 857495 | Conserved hypothetical protein | *Pyrococcus horikoshii* BAA29889 | 58/65 | No | No | No | 0 | 0 | 0 | Cyto. |
| 925374 | Pyruvate ferredoxin oxidoreductase subunit beta-2 | *Pyrococcus furiosus* X85250 | 100/100 | No | No | Yes | 0 | 0 | 0 | Cyto. |
| 926380 | Pyruvate ferredoxin oxidoreductase subunit alpha-2 | *Pyrococcus furiosus* X85250 | 100/100 | No | Unknown | No | 1 | 0 | 0 | Cyto. |
| 928888 | 2-ketovalerate ferredoxin oxidoreductase subunit alpha-2 | *Pyrococcus furiosus* X85250 | 100/100 | No | No | No | 0 | 0 | 0 | Cyto. |
| 930439 | Pyruvate ferredoxin oxidoreductase/ 2-ketovalerate ferredoxin oxidoreductase gamma subunit | *Pyrococcus furiosus* X85250 | 100/100 | No | No | No | 0 | 0 | 0 | Cyto. |
| 987716 | Methionyl-tRNA synthetase | *Mb. thermoautotrophicum* O26687 | 40/59 | No | No | No | 0 | 0 | 1 | Cyto. |
| 996088 | Conserved hypothetical protein | *Pyrococcus abyssi* CAB50022 | 62/84 | No | No | No | 0 | 0 | 0 | Cyto. |
| 1132938 | Conserved hypothetical protein | *Pyrococcus horikoshii* BAA29300 | 38/55 | No | No | No | 0 | 0 | 0 | Cyto. |
| 1197153 | Translation initiation factor (eIF-5A) | *Neurospora crassa* P38672 | 31/55 | No | No | No | 0 | 0 | 0 | Cyto. |
| 1199768 | Cystathionine gamma-lyase (gamma-cystathionase) | *Bacillus subtilus* CAB14667 | 37/55 | No | No | No | 0 | 0 | 0 | Cyto. |
| 1210188 | Superoxide reductase | *Pyrococcus furiosus* AAF03227 | 100/100 | No | No | No | 0 | 0 | 0 | Cyto. |
| 1210814 | Rubrerythrin | *Pyrococcus furiosus* AAF03227 | 100/100 | No | No | No | 0 | 0 | 0 | Cyto. |
| 1308192 | Phosphoglycerate dehydrogenase | *Methanococcus jannaschii* Q58424 | 31/39 | No | No | No | 0 | 0 | 0 | Cyto. |
| 1368790 | L-asparaginase (L-asparagine aminohydrolase) | *Bacillus subtilus* P26900 | 35/52 | No | No | No | 0 | 0 | 0 | Cyto. |
| 1425928 | Ethylene-inducible protein homolog | *Emericella nidulans* AF133101 | 54/65 | No | No | No | 0 | 0 | 0 | Cyto. |

Copyright © 2001 John Wiley & Sons, Ltd.

Table 2. Continued

| ORF # | *Pyrococcus furiosus* Genome Annotation | Closest Characterized Homolog (except for conserved hypotheticals) | Id./Sim. (%/%) | Signal Peptide: SignalP | Signal Peptide: TargetP | Signal Peptide: SOSUI | Pred. # of Transmem. Seg.: TSEG | Pred. # of Transmem. Seg.: SOSUI | Pred. # of Transmem. Seg.: PRED-TMR2 | Consensus Pred. |
|---|---|---|---|---|---|---|---|---|---|---|
| 1509633 | Myo-inositol-1-phosphate synthetase (Ino1) | *Drosophila melanogaster* AAD02819 | 16/29 | No | No | Yes | 0 | 0 | 0 | Cyto. |
| 1548241 | Imidazoleglycerol-phosphate synthetase, cyclase subunit (HisF) | *Azospirillum brasilense* P26721 | 57/74 | Yes | No | No | 0 | 0 | 0 | Cyto. |
| 1562997 | Putative 3-isopropylmalate dehydratase large subunit | *Aspergillus nidulans* Q92412 | 38/56 | No | No | No | 1 | 1 | 1 | Cyto. |
| 1692502 | Conserved hypothetical protein | *Pyrococcus horikoshii* D71189 | 89/93 | No | No | No | 0 | 0 | 0 | Cyto. |
| 1721830 | Conserved hypothetical protein | *Pyrococcus abyssi* CAB49302 | 91/95 | No | No | No | 0 | 0 | 0 | Cyto. |
| 1782302 | Conserved hypothetical protein | *Pyrococcus abyssi* F75216 | 77/86 | No | No | No | 0 | 0 | 0 | Cyto. |
| 1825269 | Thermosome, single subunit | *Desulfurococcus* sp. SY AAB35235 | 87/90 | No | No | No | 0 | 0 | 0 | Cyto. |
| 1889233 | Hypothetical protein | - | - | No | No | No | 0 | 0 | 0 | Cyto. |
| | | | Totals: | 1 (2.9%) | 0 (0%) | 3 (8.8%) | 2 (5.9%) | 1 (2.9%) | 2 (5.9%) | 0 (0%) |

with purified *P. furiosus* proteins on two-dimensional electrophoresis gels (Giometti *et al.*, 1995). The present work, however, represents the first broad-based membrane-protein identification from a hyperthermophilic archaeon. The most abundant membrane proteins in *P. furiosus* within the pI and size ranges analyzed appear to be associated with binding proteins for either peptides or maltose (Figure 1). This is consistent with the fact that this organism uses such compounds as its primary carbon source. Some of the *P. furiosus* membrane proteins did not resolve well on two-dimensional gels, especially in the first dimension, indicating that specialize solubilization conditions are needed to optimize the resolution of hyperthermophilic membrane proteins as has been observed for numerous mesophilic organisms as well (Chevallet *et al.*, 1998). Membrane-spanning proteins are generally perceived as containing hydrophobic $\alpha$-helices of $>20$ residues, but this model excludes integral membrane proteins that are highly polar, lack hydrophobic segments, and consist predominantly of $\beta$ structure (Cowan and Rosenbusch, 1994). This latter family of proteins consists primarily of porins, which are gated channels across the membrane that facilitate the diffusion of small polar solutes. Further analysis of membrane-associated proteins of *P. furiosus* predicted by the programs to be cytoplasmic suggests that two of these proteins (Pf159491 and Pf1871822) may be membrane proteins consisting primarily of $\beta$-structure (Figure 2). The Pf1871822 protein, annotated as a conserved hypothetical protein, is a $S^{\circ}$-responsive protein that dramatically increases in relative abundance when cultures are grown with $S^{\circ}$ (J. Holden and M. Adams, unpublished data; Schut *et al.*, submitted). The protein is repeatedly found in the membrane fraction using the fractionation methods described herein. Hydrophilicity, transmembrane, amphiphilic helix and sheet, and secondary structure models predict that the protein is mostly transmembrane and $\beta$-sheet structure (Figure 2A). The protein contains high concentrations of alanine, proline and glycine, which are highly abundant in porin $\beta$-barrel proteins (Li *et al.*, 1996). The product of Pf159491, another conserved hypothetical protein, may also be a $\beta$-structure membrane protein, as it is predicted to be predominantly transmembrane with up to 14 $\beta$-sheet structures, and contains moderately high concentrations of the same three amino acid residues (Figure 2B).

Copyright © 2001 John Wiley & Sons, Ltd.

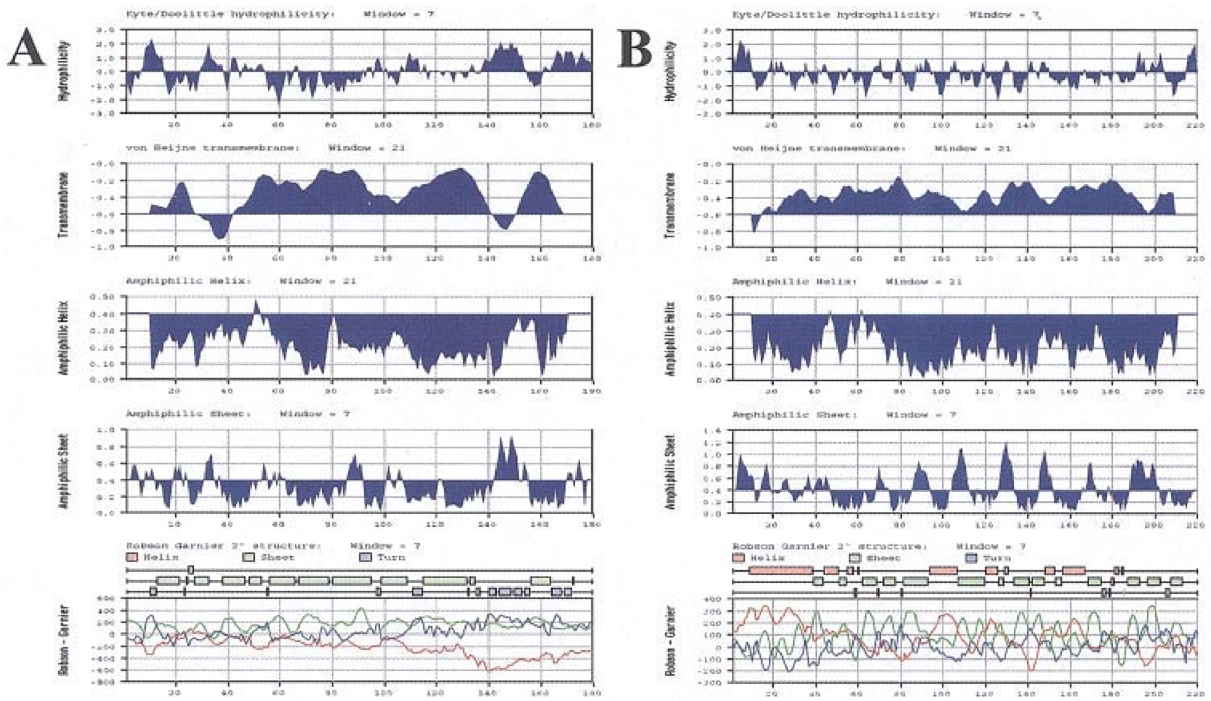

**Figure 2.** Predictions of Kyte/Doolittle hydrophily, von Heijne transmembranes, amphiphilic helices and sheets, and Robson-Garnier secondary structure for the putative membrane proteins encoded by Pf1871822 (A) and Pf159491 (B). The predictions are derived from the MacVector v7.0 sequence analysis software

The product of Pf189210 is homologous to subunit E of a characterized membrane-bound V-type ATPase from the hyperthermophilic archaeon *Desulfurococcus* sp. SY (Shibui *et al.*, 1997). Although predicted to be a cytoplasmic protein by the programs used, this protein was among the proteins identified in the membrane fraction after separation by 2DE. Two other subunits of this ATPase (subunits K from Pf188579 and C from Pf190329), predicted to be membranous, were also identified in the membrane fraction. Therefore, the Pf189210 protein may form a membrane complex with the Pf188579 and Pf190329 proteins. Two *P. furiosus* operons encoding the characterized membrane hydrogenase and the Nuo homologs also contain a mixture of known and presumed membrane and cytoplasmic proteins (Sapra *et al.*, 2000; R. Sapra and M. Adams, unpublished data). The 'cytoplasmic' components of these complexes may also be associated with the membrane and in this case the prediction that they are cytoplasmic proteins by the programs that were used herein would be inaccurate.

The Pf1074447 protein, a conserved hypothetical, is predicted to be part of a 13 ORF operon consisting entirely of conserved hypothetical ORFs (assuming an operon contains two or more ORFs that are separated by less than 16 nucleotides). Using the membrane prediction programs, at least one of the other ORFs in this operon (Pf1071624) encodes for a membrane protein. Therefore, the Pf1074447 protein could potentially form a complex with the Pf1071624 protein in the membranes. None of the ORFs encoding the other cytoplasmic-predicted proteins from the membrane fraction were in putative operons with a predicted membrane-protein-encoding ORF. However, these proteins may complex with other membrane proteins or have a unique membrane protein structure. Three of the proteins identified in the membrane fraction of *P. furiosus* that were predicted to be cytoplasmic (Pf336067, Pf1459639 and Pf1825269) show homology to known cytoplasmic proteins (i.e., inosine monophosphate dehydrogenase, DNA directed RNA polymerase subunit B, and thermosome, respectively). Pf336067 and Pf1825269 were also identified in the cytoplasmic protein fraction (Table 2). It seems likely that they are cytoplasmic

Copyright © 2001 John Wiley & Sons, Ltd. 

proteins that are contaminating the membrane fraction. For example, the Pf1825269 protein that is highly homologous to a known hyperthermophilic archaeal chaperonin may have attached to membrane proteins that denatured during the fractionation process. Nouwens *et al.* (2000) did not find any cytoplasmic contaminants in their membrane protein fractions. The presence of cytoplasmic contaminants in our membrane protein preparations may be explained by our method of protein detection and identification (i.e., μ-LC-MS/MS and MALDI-TOF), which is significantly more sensitive than their method (MALDI-TOF only).

In two instances, there was agreement among signal peptide predictions and among transmembrane sequence predictions, but disagreement between the two sets of predictions. For the Pf1186264 protein from the membrane fraction, no signal peptide was recognized, but one transmembrane sequence was predicted. The Pf1186264 protein shows 30% identity with the *P. abyssi* protein D75122 when analyzed across the entire protein, but aligns with the C-terminal end of the protein with 53% identity and 71% similarity. Pf1184685 is 344 bp upstream of Pf1186264 and aligns with the N-terminal end of the same *P. abyssi* protein with 29% identity and 51% similarity. The same trends were observed when these two ORF products were compared with *P. horikoshii* protein B71009. The Pf1184685 protein contains a putative signal peptide sequence. Therefore, there appears to be a frameshift in the *P. furiosus* nucleotide sequence and Pf1184685 and Pf1186264 may belong to the same ORF. For the Pf1562997 protein from the cytoplasmic fraction, the predicted transmembrane sequence is between residues 115 and 137 (out of 380) and most likely does not represent a membrane protein. These results underscore the importance of using the consensus of multiple models coupled with other analyses on questionable ORF product predictions to accurately predict protein subcellular locations.

Using a consensus of all six membrane protein prediction models, it was estimated that 24% of the ORF's in the *P. furiosus* genome encode for a membrane protein. This value is similar to those predicted for other hyperthermophilic archaea for which complete genome sequences are available, which include *P. horikoshii*, *Archaeoglobus fulgidus*, and *Methanococcus jannaschii* (Kihara and Kanehisa, 2000; Mitaku *et al.*, 1999; Pasquier and Hamodrakas, 1999; Paulsen *et al.*, 2000; Wallin and von Heijne, 1998). For each of these predictions only one analysis program was used, and it was predicted that 14–23% of the ORF's in their genome encode membrane proteins. Based on our sequence analyses of proteins identified by proteomics, it appears that the most accurate estimate of subcellular location occurs when a consensus is made between the six programs.

The signal peptide and transmembrane α-helix programs used in this study generally predict accurately the subcellular location of the proteins identified by proteomics in membrane and cytoplasmic fractions when used in a consensus fashion. Therefore, they are useful for "first pass" predictions of membrane proteins, although they do not detect membrane proteins that consist of β-sheets rather than α-helices, nor do they recognize 'cytoplasmic' proteins that form complexes with membrane proteins. Therefore, careful follow-up analyses are necessary for a complete census of membrane proteins. Nielsen *et al.* (1999) stated that much more is known about membrane proteins from organisms other than archaea, and that their analyses with *M. jannaschii* suggest unique membrane proteins structures may exist in this organism, as well as in other archaea. Very little is known about membrane proteins in *P. furiosus*. Accurate membrane protein prediction models are vital for research on *P. furiosus*, especially for on-going structural genomic efforts and other analyses involving the membrane.

### Acknowledgements

We thank A. Lal Menon, G. Schut, and F. Jenney for their laboratory assistance and helpful advice for this project. This research was funded by grants from the National Science Foundation (MCB 9904624, MCB 9809060, and BES-0004257 to MWWA) and the US Department of Energy Office of Biological and Environmental Research (FG05-95ER20175 to MWWA and contract No. W-31-109-ENG-38 to CSG, JRY, and MWWA).

### References

Adams MWW. 1999. The biochemical diversity of life near and above 100°C in marine environments. *J Appl Microbiol Symp Suppl.* **85**: 108S–117S.

Adams MWW, Holden JF, Lal Menon A, *et al.* 2001. Key role for sulfur in peptide metabolism and in regulation of three hydrogenases in the hyperthermophilic archaeon *Pyrococcus furiosus*. *J Bacteriol* **183**: 716–724.

Copyright © 2001 John Wiley & Sons, Ltd. 

Anderson NG, Anderson NL. 1978a. Analytical techniques for cell fractions. XXI. Two-dimensional analysis of serum and tissue proteins: multiple isoelectric focusing. *Anal Biochem* **85**: 331–340.

Anderson NL, Anderson NG. 1978b. Analytical techniques for cell fractions. XXI. Two-dimensional analysis of serum and tissue proteins: multiple gradient slab-gel electrophoresis. *Anal Biochem* **85**: 341–354.

Andersson H, Bakker E, von Heijne G. 1992. Different positively charged amino acids have similar effects on the topology of a polytopic transmembrane protein in *Escherichia coli. J Biol Chem* **267**: 1491–1495.

Bradford MM. 1976. A rapid and sensitive method for the quantitation of microgram quantities of protein utilizing the principle of protein-dye binding. *Anal Biochem* **72**: 248–254.

Burley SK. 2000. An overview of structural genomics. *Nature Structural Biol* **7**: 932–934.

Chevallet M, Santoni V, Poinas A, *et al.* 1998. New zwitterionic detergents improve the analysis of membrane proteins by two-dimensional electrophoresis. *Electrophoresis* **19**: 1901–1909.

Cordwell SJ, Nouwens AS, Verrills NM, Basseal DJ, Walsh BJ. 2000. Subproteomics based upon protein cellular location and relative solubilities in conjunction with composite two-dimensional electrophoresis gels. *Electrophoresis* **21**: 1094–1103.

Cowan SW, Rosenbusch JP. 1994. Folding pattern diversity of integral membrane proteins. *Science* **264**: 914–916.

Dove A. 1999. Proteomics: translating genomics into products? *Nature Biotechnol* **17**: 233–236.

Emanuelsson O, Nielsen H, Brunak S, von Heijne G. 2000. Predicting subcellular localization of proteins based on their N-terminal amino acid sequence. *J Mol Biol* **300**: 1005–1016.

Eng JK, McCormack AL, Yates JR III. 1994. An approach to correlate mass spectral data of peptides with amino acid sequences in a protein database. *J Am Soc Mass Spectrom* **5**: 976–989.

Fiala G, Stetter KO. 1986. *Pyrococcus furiosus* sp. nov. represents a novel genus of marine heterotrophic archaebacteria growing optimally at 100°C. *Arch Microbiol* **145**: 56–61.

Gatlin CL, Kleemann GR, Hays LG, Link AJ, Yates JR III. 1998. Protein identification at the low femtomole level from silver-stained gels using a new fritless electrospray interface for liquid chromatography-microspray and nanospray mass spectrometry. *Anal Biochem* **263**: 93–101.

Giometti CS, Gemmell MA, Nance SL, Tollaksen SL, Taylor J. 1987. Detection of heritable mutations as quantitative changes in protein expression. *J Biol Chem* **262**: 12764–12767.

Giometti CS, Gemmell MA, Tollaksen SL, Taylor J. 1991. Quantitation of human leukocyte proteins after silver staining: a study with two-dimensional electrophoresis. *Electrophoresis* **12**: 536–543.

Giometti CS, Tollaksen SL, Mukund S, *et al.* 1995. Two-dimensional gel electrophoresis mapping of proteins isolated from the hyperthermophile *Pyrococcus furiosus. J Chromatogr A* **698**: 341–349.

Hirokawa T, Boon-Chieng S, Mitaku S. 1998. SOSUI: classification and secondary structure prediction system for membrane proteins. *Bioinformatics* **14**: 378–379.

Kihara D, Shimizu T, Kanehisa M. 1998. Prediction of membrane proteins based on classification of transmembrane segments. *Protein Eng* **11**: 961–970.

Kihara D, Kanehisa M. 2000. Tandem clusters of membrane proteins in complete genome sequences. *Genome Res* **10**: 731–743.

Li S-C, Goto NK, Williams KA, Deber CM. 1996. α-helical, but not β-sheet, propensity of proline is determined by peptide environment. *Proc Natl, Acad Sci U S A* **93**: 6676–6681.

Link AJ, Hays LG, Carmack EB, Yates JR III. 1997. Identifying the major proteome components of *Haemophilus influenzae* type-strain NCTC 8143. *Electrophoresis* **18**: 1314–1334.

Mitaku S, Ono M, Hirokawa T, Boon-Chieng S, Sonoyama M. 1999. Proportion of membrane proteins in proteomes of 15 single-cell organisms analyzed by the SOSUI prediction system. *Biophys Chem* **82**: 165–171.

Nielsen H, Engelbrecht J, Brunak S, von Heijne G. 1997. Identification of prokaryotic and eukaryotic signal peptides and prediction of their cleavage sites. *Protein Eng* **10**: 1–6.

Nielsen H, Brunak S, von Heijne G. 1999. Machine learning approaches for the prediction of signal peptides and other protein sorting signals. *Protein Eng* **12**: 3–9.

Nouwens A, Cordwell SJ, Larsen MR, *et al.* 2000. Complementing genomics with proteomics: the membrane subproteome of *Pseudomonas aeruginosa* PAO1. *Electrophoresis* **21**: 3797–3809.

O'Farrell PH. 1975. High-resolution two-dimensional electrophoresis of proteins. *J Biol Chem.* **250**: 4007–4021.

Pasquier C, Hamodrakas SJ. 1999. An hierarchical artificial neural network system for the classification of transmembrane proteins. *Protein Eng* **12**: 631–634.

Paulsen I, Nguyen L, Sliwinski MK, Rabus R, Saier MH Jr. 2000. Microbial genome analyses: comparative transport capabilities in eighteen prokaryotes. *J Mol Biol* **301**: 75–100.

Ramagli LS, Rodriguez LV. 1985. Quantitation of microgram amounts of protein in two-dimensional polyacrylamide gel electrophoresis sample buffer. *Electrophoresis* **6**: 559–563.

Robb FT, Park J-B, Adams MWW. 1992. Characterization of an extremely thermostable glutamate dehydrogenase: a key enzyme in the primary metabolism of the hyperthermophilic archaebacterium, *Pyrococcus furiosus. Biochim Biophys Acta* **1120**: 267–272.

Robb FT, Maeder DL, Brown JR, *et al.* 2001. Genomic sequence of hyperthermophile, *Pyrococcus furiosus*: implications for physiology and enzymology. *Methods Enzymol* **330**: 134–157.

Sapra R, Verhagen MFJM, Adams MWW. 2000. Purification and characterization of a membrane-bound hydrogenase from the hyperthermophilic archaeon *Pyrococcus furiosus. J Bacteriol* **182**: 3423–3428.

Schut GJ, Zhou J, Adams MWW. DNA microarray analysis of the hyperthermophilic archaeon *Pyrococcus furiosus*: evidence for a new type of sulfur-reducing enzyme complex. *J Bacteriol* (submitted 5/01).

Shibui H, Hamamoto T, Yohda M, Kagawa Y. 1997. The stabilizing residues and the functional domains in the hyperthermophilic V-ATPase of *Desulfurococcus. Biochem Biophys Res Comm* **234**: 341–345.

Southern E, Mir K, Shchepinov M. 1999. Molecular interactions on microarrays. *Nature Genet* **21**: 5–9.

Stetter KO. 1999. Extremophiles and their adaptation to hot environments. *FEBS Lett* **452**: 22–25.

Copyright © 2001 John Wiley & Sons, Ltd. 

Verhagen MFJM, Lal Menon A, Schut GJ, Adams MWW. 2001. *Pyrococcus furiosus*: large-scale cultivation and enzyme purification. *Methods Enzymol* **330**: 25–30.

Wallin E, von Heijne G. 1998. Genome-wide analysis of integral membrane proteins from eubacterial, archaean, and eukaryotic organisms. *Protein Sci* **7**: 1029–1038.

*The*
**Genomics**
*Website at Wiley*

**www.wiley.co.uk/genomics**

The **Genomics** website at Wiley is a **DYNAMIC** resource for the genomics community, offering **FREE** special feature articles and new information **EACH MONTH**.

Find out more about our new journals *Comparative and Functional Genomics,* and *Proteomics.*

Visit the **Library** for hot books in Genomics, Bioinformatics, Molecular Genetics and more.

Click on **Journals** for information on all our up-to-the minute journals, including: *Genesis, Bioessays, Journal of Mass Spectrometry, Gene Function and Disease, Human Mutation, Genes, Chromosomes and Cancer* and the *Journal of Gene Medicine.*

Let the **Genomics** website at Wiley be your **guide** to genomics-related web sites, manufacturers and suppliers, and a calendar of conferences.

WILEY

2380

Copyright © 2001 John Wiley & Sons, Ltd. 
